# Identification of E2 with improved secretion and immunogenicity against CSFV in piglets

**DOI:** 10.1186/s12866-020-1713-2

**Published:** 2020-02-04

**Authors:** Huiling Xu, Yanli Wang, Guangwei Han, Weihuan Fang, Fang He

**Affiliations:** 1 Institute of Preventive Veterinary Medicine, College of Animal Sciences of Zhejiang University, 866 Yuhangtang road, Hangzhou, 310058 China; 2 Zhejiang Provincial Key Laboratory of Preventive Veterinary Medicine, Hangzhou, China

**Keywords:** Classical swine fever virus, Novel signal peptide, SPZJ-E2ZJ, Subunit vaccine, Protective immunity

## Abstract

**Background:**

Outbreaks of Classical swine fever virus (CSFV) cause significant economic losses in the swine industry. Vaccination is the major method to prevent and control the disease. As live attenuated vaccines fail to elicit differentiable immunity between infected and vaccinated animals, subunit vaccine was considered as an alternative candidate to prevent and eradicate CSFV. Subunit vaccines present advantages in DIVA immunogenicity and safety. The technology was limited due to the low yield and the high cost with multiple and large doses. The native E2 signal peptide has not been well defined before. Here, the aim of this study is to develop a cost-effective and efficacious E2 vaccine candidate against CSFV with signal peptide and E2 sequence selection.

**Results:**

A novel CSFV E2 sequence (E2ZJ) was identified from an epidemic strain of Zhejiang for outstanding secretion in baculovirus and enhanced immunogenicity. E2 secretion induced with the selected signal peptide, SPZJ (SP23), increase at least 50% as compared to any other signal peptides tested. Besides, unique antigenic features were identified in E2ZJ. As indicated with immunized sera in IFA against CSFV infection, E2ZJ elicited CSFV antibodies at the earlier stage than other E2 types tested in mice. Moreover, higher level of neutralizing and CSFV antibodies against CSFV with E2ZJ was detected than other E2s with the same dosage at 28 dpi. Further, E2ZJ successfully elicited neutralizing immunity in piglets. A single dose of 5 μg of E2ZJ was sufficient to induce protective antibodies against CSFV in piglets and provided 100% protection against lethal virus challenge.

**Conclusions:**

Our studies provide evidence that E2ZJ guided by a novel E2 signal peptide (SPZJ) was efficiently secreted and presented significantly improved immunogenicity than conventional E2 vaccines. Moreover, a single dose of 5 μg E2ZJ is efficacious against CSFV in piglets.

## Background

Classical Swine Fever (CSF) is a highly contagious viral disease which is economically important to the pig industry worldwide [1]. It is endemic in Asia, some areas of Central and South America and in many Eastern European countries with sporadic occurrence in Western Europe [2, 3]. Classical Swine Fever Virus (CSFV) has three major genetic groups, namely, Groups 1, 2, and 3, each comprising three to four subgenotypes (1.1 to 1.4, 2.1 to 2.3, and 3.1 to 3.4) [4, 5]. Genotypes 1and 2 have been identified in China [6, 7]. The disease can cause several clinical signs, including fever, loss of appetite, weakness and conjunctivitis, which were often accompanied by death within 10–20 days of infection [8]. The etiological agent, Classical Swine Fever Virus (CSFV), belongs to the Pestivirus genus within the family Flaviviridae [9]. CSFV genome contains a single open reading frame that encodes a polyprotein which is processed into 12 proteins, including Npro, C, Erns, E1, E2, p7, NS2, NS3, NS4A, NS4B, NS5A and NS5B [10, 11]. Previous studies revealed that a critical area in E2 is required for CSFV replication in SK6 cells between protein residues 136–156 [12]. Besides, the structural protein E2 is a key determinant for viral entry and immunity [13]. It has been well established that CSFV E2 protein is the major protective antigen which elicits neutralizing antibodies that make it important for producing subunit vaccines against CSFV [14, 15].

Currently, vaccination is the widely used strategy to prevent CSF. Most vaccines available against CSFV are live attenuated vaccines (LAVs). So far, commercially available vaccines mainly consist of live attenuated CSFV of cell line origin or rabbit tissue origin, derived from the commonly used C-strain. These live attenuated vaccines have outstanding efficacy and safety but lack a serological concept of differentiating infected from vaccinated animals (DIVA) thus hampering CSF eradication and cause concern in animal welfare when live rabbits are exploited in vaccine production [16, 17]. Thus, in these years, efforts have been made on E2 based subunit vaccines for alternative option against CSFV. E2 subunit vaccines have been confirmed to induce sufficient CSFV specific antibodies and provide complete protection against homologous CSFV in rabbits [18] and pigs [19, 20]. Commercial CSFV E2 subunit vaccine Porcilis® Pesti derived from genotype 1 fail to elicit complete protection against heterologous strains of genotype 2.1 [18], while by booster immunization another commercial vaccine TWJ-E2® (genotype 1) was reported to provide complete protection against heterologous strains of genotype 2 [21]. However, these subunit vaccines usually require large multiple doses to induce the comparable CSFV-specific protective immunity as C-strain LAVs [22]. And there is a delay in the induction of the protective antibody response upon vaccination [8, 23]. Besides, due to the low yield of E2 in a soluble and correct-folded form, the wide application of E2 in CSFV vaccine market is limited [24]. Therefore, the enhancement of soluble E2 expression with effective immunogenicity is the corner stone for this CSFV vaccine to be one of the most efficacious and practical strategies against CSFV in future.

Subunit vaccine immunogenicity, including cross-immunogenicity, is primarily determined by the protein sequence and the expression system. Phylogeny and antigenicity characterization will be usefully to identify targets with outstanding immunogenicity. Meanwhile, recombinant baculovirus expression system produces target proteins with correct protein structure and post-translational modifications such as protein glycosylation and disulfide bonds formation as their natural conformation [25, 26], which leads to efficacious immunogenicity of vaccines. In baculovirus, soluble E2 secretion in culture supernatant depends on a suitable signal peptide [27]. However, to the date, the exact sequence of the signal peptide for soluble products has not been reported from native E2. And the secretion efficiency and the immunogenicity of E2 have not been well optimized to meet the needs in vaccine production.

To improve the efficacy and wide application of E2 vaccine against CSFV, E2s from different CSFV strains were expressed and compared in this study. A novel signal peptide SPZJ successfully induces higher E2 expression in cell culture supernatant than any other constructs tested. The improved immune response elicited by E2ZJ protein is detected in Immunofluorescence assay (IFA) and neutralization test as compared to E2HZ and E2C. Furthermore, the protection and immunogenicity of a single dose of E2ZJ protein were evaluated in piglets against lethal CSFV challenge. Thus, SPZJ-E2ZJ is a promising candidate subunit vaccine against CSFV.

## Results

### Identification of a novel signal peptide SPZJ for robust E2 production

**Fig. 1:**
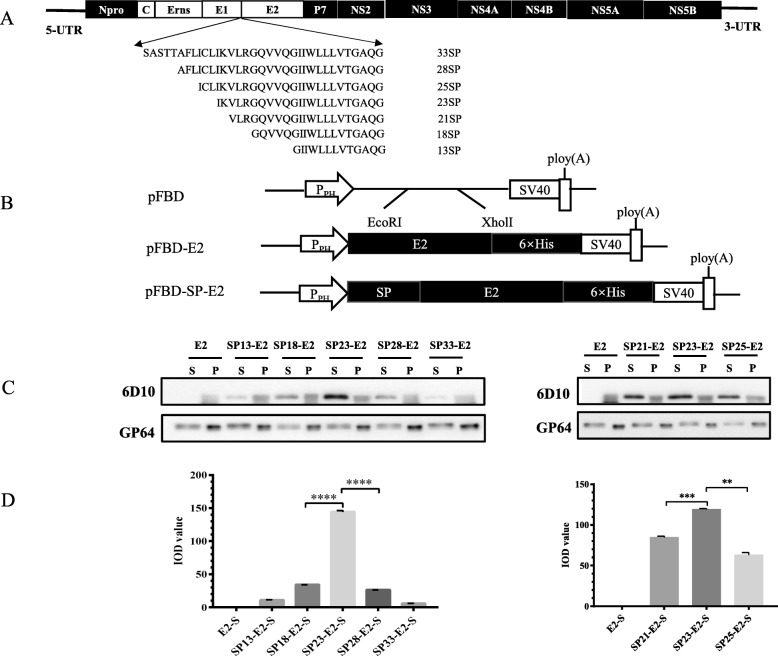
Identification of signal peptides for E2 expression in baculovirus. **a** Amino acid sequences of different truncated E2 signal peptides. **b** Schematic diagrams of different transfer plasmids for recombinant baculoviruses Ac-E2 and Ac-SP-E2. **c** E2 expression and secretion with different signal peptides detected in Western blotting. Culture supernatant (S) from cell culture and pellet (P) of High Five cells resuspended in PBS were examined with anti-E2 mAb 6D10, and with anti-Gp64 mAb as control. All samples were loaded in equal volume. **d** E2 secretion levels with different signal peptides were quantified with densitometric analysis using Gel-Pro analyzer software. The results of integral optical density (IOD) stand for protein expression levels. Experiments were performed in triplicate and data are shown as mean ± SD. Statistical significance is indicated as **(*P* < 0.01); ***(*P* < 0.001); ****(*P* < 0.0001)

**Fig. 2:**
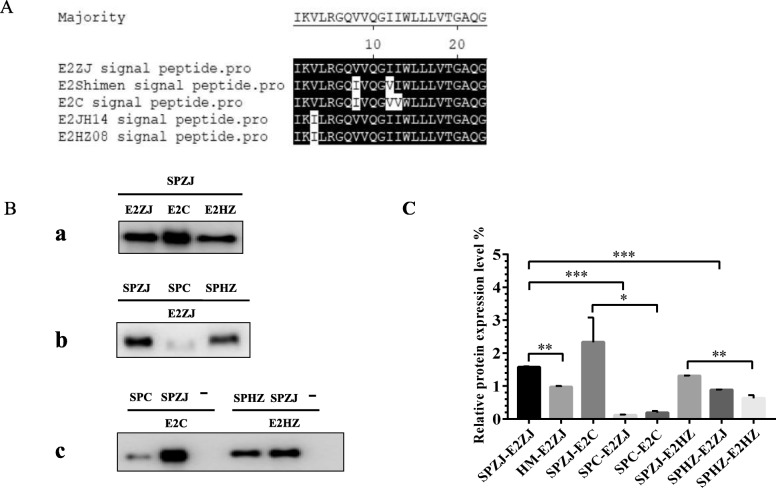
Characterization of SPZJ as an efficient E2 signal peptide. **a** Alignment of SP23s from different CSFV strains. **b** Comparison between SP23s (SPZJ, SPC and SPHZ) in secretion of different E2s. E2 proteins were purified by Ni-NTA Agarose from equal volume of baculovirus culture prepared in the same method. E2 were detected in Western blotting with anti-E2 mAb 6D10. **a**. SPZJ is active for different E2 types. **b**. Comparison of SPs in induction of ZJE2. **c**. Comparison of SPZJ with other SPs in induction of their native E2s. **c** The production of E2 with SPZJ. The relative level of E2 was determined using E2ZJ induced with honeybee melittin signal peptide (HM) as base (1.0). The yield of secreting E2 with SPZJ was compared with other signal peptides, including SPC, SPHZ and HM. Experiments were performed in triplicate and data are shown as mean ± SD. Statistical significance is indicated as *(*P* < 0.05); **(*P* < 0.01); ***(*P* < 0.001)

To identify the efficient native signal peptide for E2 secretion, E2 protein was fused to a series of truncated candidates (SP13, SP18, SP21, SP23, SP25, SP28 or SP33) individually (Fig. 1a). Then, the transfer vectors named as pFBD-E2, pFBD-SP-E2 were used to generate recombinant baculoviruses by the Bac-to-Bac system (Fig. 1b). SP23 induced higher level of E2 secretion than any other signal peptides tested (Fig. 1c, d). Sequence variation was found in SP23s among different CSFV strains, including ZJ01, HZ08 and C strain (Fig. 2a). SP23 of ZJ01 (SPZJ) was able to induce the expression of other E2s (E2C and E2HZ) as well as its native E2ZJ, indicating SPZJ is a common signal peptide for variable E2 production (Fig. 2b-a). To identify the best peptide for E2 secretion, the expression of E2 under different SP23s in baculovirus were compared (Fig. 2b-b). SPZJ was revealed to induce higher level of E2ZJ secretion than the signal peptides from HZ08 (SPHZ) and C strain (SPC). Finally, SPZJ was compared with SPHZ and SPC individually to induce the secretion of E2HZ and E2C. The secretion level of either E2HZ or E2C elicited with SPZJ is higher than the one with their native signal peptides (Fig. 2b-c), confirming SPZJ is a strong signal peptide for different E2s to secret in baculovirus. Furthermore, SPZJ induced at least 50% increase in E2 secretion as compared to conventional signal peptides, such as honeybee melittin signal peptide and SPC from C strain. Meanwhile, stronger E2 expression was induced with SPZJ than the signal peptides from SPHZ and SPC, indicating SPZJ is more efficient to guide its native E2 expression as compared with other heterologous signal peptides (Fig. 2c). In the small-scale production with shaking flasks (50 ml), E2 yield with SPZJ is up to 65 μg/ml. The yield could be further enhanced when SPZJ is applied to industrial production.

### E2ZJ elicits efficient antibody response against CSFV

**Fig. 3:**
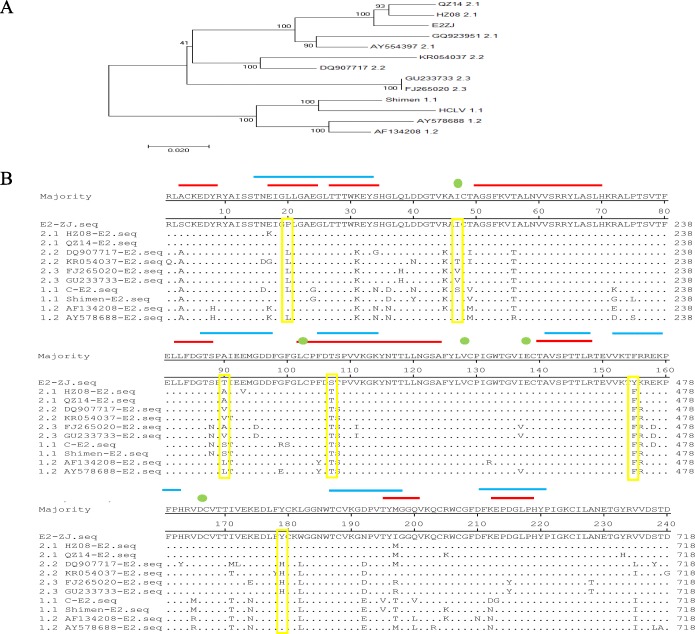
Antigenicity analysis of E2s among different genetic groups. **a** The phylogenetic tree based on CFSV E2 genes was constructed, using the neighbor-joining method. E2ZJ was classified to subgroup 2.1. **b** Alignment of E2 proteins. Antigenic regions were highlighted: identified epitopes (red lines), predicted epitopes (blue lines) and identified conformational epitopes (green dots). Characteristic amino acids of E2ZJ were labeled with yellow boxes. Sequences were aligned using MegAlign

**Fig. 4:**
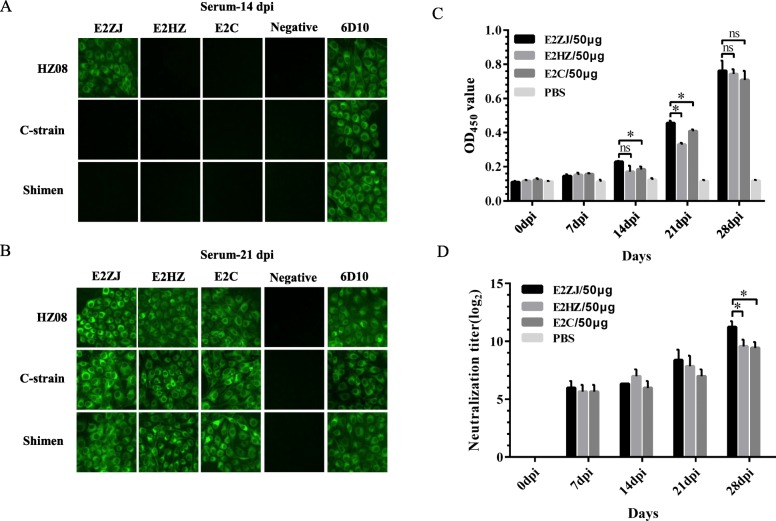
Efficient immunogenicity of E2ZJ in mice. Twenty six-week-old female BALB/c mice were randomly divided into 4 groups (A, B, C and D, *n* = 5). Groups A to C were respectively immunized twice with 50 μg of E2 (E2ZJ, E2HZ or E2C). Group D was injected with PBS as control. **a**, **b** Detection of CSFV-specific antibodies in mouse sera elicited with different E2s (E2ZJ, E2HZ and E2C) with CSFV infected cells. PK-15 cells were infected with HZ08, C-strain or Shimen at MOI of 0.1. Cells were stained respectively with diluted serum (1:200) collected at 14 dpi and 21 dpi. Negative serum was from mice without any immunization. Anti-E2 mAb 6D10 was used as controls to indicate successful infection. **c** Detection of E2-specific antibodies in mouse serum with ELISA. Diluted serum samples (1:200) after days post vaccination were determined by indirect ELISA. **d** Detection of CSFV neutralizing antibodies against HZ08 strain in mouse serum. Serum samples were collected at weekly intervals after immunization with different E2s. PBS group is the sera from mice injected with PBS only. Experiments were performed in triplicate and data are shown as mean ± SD. Statistical significance is indicated as (ns) (*P* > 0.05); *(*P* < 0.05)

E2ZJ was classified to group 2.1 based on phylogenetic tree (Fig. 3a). There are three signature mutations, T107S, F155Y and 90 T, identified in the antigenic regions of E2ZJ. Meanwhile, E2ZJ, as a strain of group 2, presents sequence features of group 1 as well, such as 20P and 179P (Fig. 3b). This epitope-chimera-like characteristic potentially improve the cross immunogenicity [28]. Thus, the immunogenicity of three recombinant E2s (E2ZJ, E2HZ, E2C) was primarily tested in mice. As shown in IFA with CSFV infected cells and immunized sera 1:200 diluted, specific antibodies against the same genotype 2 were observed in the group vaccinated with E2ZJ as early as 14 dpi while no antibodies were detected in groups with other E2 types (Fig. 4a). The other E2 vaccinated groups showed positive CSFV antibody response at 21 dpi while E2ZJ elicited significant heterologous immunogenicity against genotype 1 (Fig. 4b). Further, in ELISA, E2 antibody level in all vaccinated groups started to rise up at 14 dpi. However, higher E2 antibody level against both two genotypes was observed in the group of E2ZJ as compared to other groups from 14 dpi to 28 dpi (Fig. 4c). Similarly, E2ZJ elicited significantly higher level of CSFV neutralizing antibodies than other E2 types. At 28 dpi, the neutralization titer of E2ZJ was up to 2000 while the titers from other E2 groups were less than 500. Neither CSFV-specific antibodies nor neutralizing antibodies were detected in PBS group (Fig. 4d). These findings indicated that E2ZJ presents improved protective immunogenicity than other candidates against different E2s.

### **Single dose of E2ZJ** stimulates protective antibody response in piglets against CSFV

**Fig. 5:**
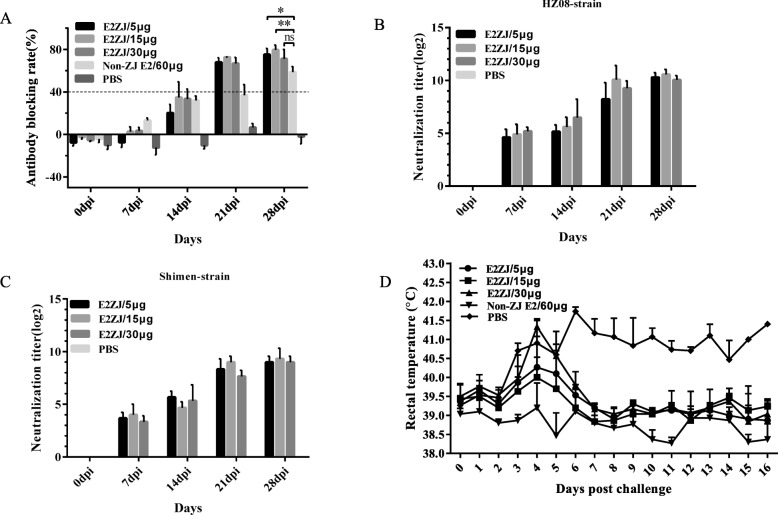
Protective immunogenicity of E2ZJ in piglets. Twenty-five piglets were randomly divided into 5 groups (E, F, G, H and L, *n* = 5). Groups E to G were respectively immunized with a single dose of E2ZJ at 5 μg, 15 μg or 30 μg. Group H was immunized with a single dose of 60 μg of Non-ZJ E2. Group L was injected with PBS as control. **a** Detection of CSFV-specific antibodies in pig sera with ELISA. Serum samples from pigs immunized with different antigens after days post vaccination were collected to detect the CSFV-specific antibodies using the IDEXX HerdChek® CSFV Antibody Test Kit. **b**, **c** Detection of CSFV neutralizing antibody in pig sera. Neutralization titer was determined against 100 TCID_50_ of CSFV HZ08 strain (**b**) and Shimen strain (**c**). PBS: the group was injected with PBS only. **d** Rectal temperatures of the piglets after challenge with CSFV Shimen strain. Non-ZJ E2 was a CSFV E2 candidate vaccine expressed in HEK293 expression system other than baculovirus. Experiments were performed in triplicate and data are shown as mean ± SD. Statistical significance is indicated as (ns) (*P* > 0.05): *(*P* < 0.05); **(*P* < 0.01)

The efficacy of E2ZJ to elicit the protective antibody response was further evaluated in piglets. Twenty-five piglets were individually vaccinated with different dosages of E2ZJ (5 μg, 15 μg or 30 μg) to determine the minimal effective dose for the complete protection against CSFV. In competitive ELISA for CSFV antibody with a commercial kit, at 14 dpi, the mean antibody blocking rates of piglets vaccinated with 15 μg and 30 μg of E2ZJ were higher than the cut-off value of 40% though the group of 5 μg was less than 25%. Meanwhile, in the group of non-ZJ E2 at the dose of 60 μg, average antibody blocking rate was less than 40%. Non-ZJ E2 was a CSFV E2 vaccine candidate expressed in HEK293 expression system other than baculovirus expression system. At 21 dpi, the blocking rates were higher than 40% as positive in all vaccinated groups, including the groups of 5 μg of E2ZJ. The antibody response of all E2ZJ groups increased at 28 dpi at the similar level, which exceeded the group of non-ZJ E2. No CSFV-specific antibodies were detected in PBS group (Fig. 5a). CSFV neutralizing antibody response with E2ZJ was evaluated against either a pandemic strain HZ08 (Fig. 5b) or the challenge strain Shimen (Fig. 5c). At 7 dpi, CSFV neutralizing antibodies were initially detected in all E2 vaccinated groups, including the group of 5 μg, but not in PBS group. Meanwhile, the neutralizing antibodies titers in 5 μg, 15 μg or 30 μg groups at 28 dpi were of 1:1286, 1:1564, 1:1089 against HZ08 and 1:533, 1:746, 1:533 against Shimen strain respectively. Neutralization antibody titers against HZ08 strain were higher than those against Shimen strain. PBS group showed no neutralizing antibodies all the time.

### **Low-dose** immunization **with E2ZJ** protected piglets from CSFV lethal challenge

Further, the protection with E2ZJ against CSFV lethal challenge was studied based on clinical symptoms and pathologic presentation. From 3 days after challenge infection, the control PBS group presented cute febrile response (41–41.8 °C) and other clinical symptoms including diarrhea and anorexia. Four of 5 infected piglets in the PBS group died at 15 dpc (days post challenge). All piglets immunized with E2, including the group of 5 μg E2ZJ, did not develop any symptoms during the whole experiment, except that one pig from the group with 30 μg showed temporary mild fever response (40.3–41.5 °C) at 4 dpc and recovered at 6 dpc (Fig. 5d).

**Fig. 6:**
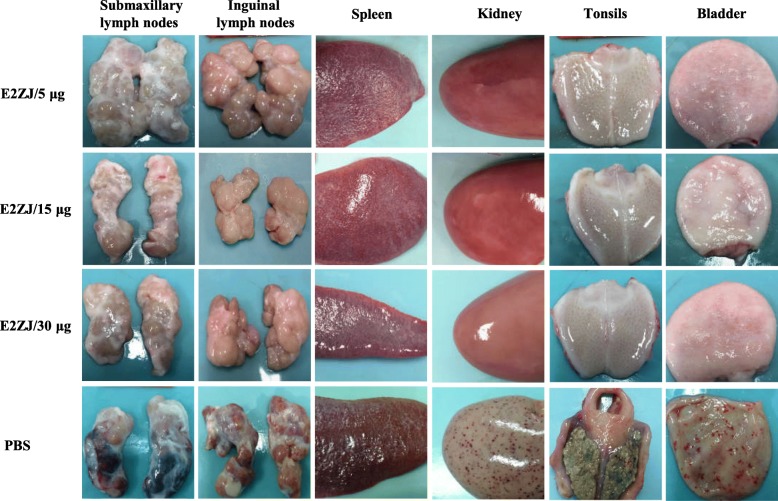
Representative pathological examination of immunized pigs challenged with CSFV Shimen strain. Five groups of pigs (*n* = 5) were immunized and challenged as described. At 16 dpc, all survived pigs were euthanized and different tissues (spleen, kidney, tonsils, lymph nodes and bladder) were collected for pathological examinations

**Fig. 7:**
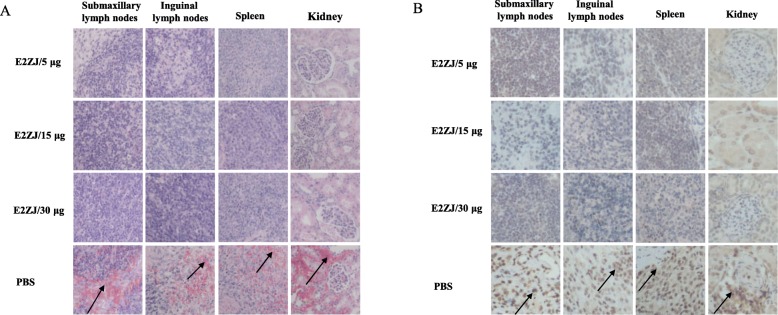
Representative histopathological and immunohistochemistrical examination of immunized pigs challenged with CSFV Shimen strain. Various tissues (spleen, kidney, submandibular lymphatic nodes and inguinal lymph nodes) were collected for histopathological evaluation (**a**) and immunohistochemistry study (**b**) with CSFV polyserum as primary antibody. The pathological lesion and CSFV specific signals were indicated with arrows

At 16 dpc, all the piglets were euthanized and subjected to pathological and histopathological examination. Non-vaccinated PBS group appeared severe clinical lesions in different organs, including defusing hemorrhage in the kidney and bladder and necrosis in the tonsils. Besides, splenic infarcts and petechia in submaxillary and inguinal lymph nodes were observed in the PBS group. In contrast, no lesion was detected in any organ tested of all E2ZJ vaccinated groups (Fig. 6). Similar severe histopathological lesions in the control group were shown in H&E (Fig. 7a), including depleted lymphoid follicles and hemorrhages (arrow) in the lymph nodes, diffusing hemorrhages (arrow) throughout splenic parenchyma and interstitial spaces of kidney.

CSFV viral antigens in tissues were examined by immunohistochemistry with anti-CSFV polyserum. As shown in Fig. 7b, CSFV positive signals (arrow) were observed in tissues of PBS group but not in the groups vaccinated with any dose of E2ZJ, not even with the dose of 5 μg. These results indicated that a single dose of 5 μg of E2ZJ in piglets successfully prevented CSFV infection relying on the efficient elicitation of protective antibody response against CSFV.

## Discussion

Outbreaks of CSFV cause significant economic losses in the swine industry. E2 based subunit vaccines have been shown to be a promising strategy for the control and eradication of this disease, as live attenuated vaccines fail to elicit differentiable immunity between infected and vaccinated animals [29, 30]. In contrast to prokaryotic systems, baculovirus infected insect cells produce recombinant proteins with correct 3D structure and glycosylation as the original eukaryotic system, which leads to the proper biological function and immunogenicity in protein products, making them functional vaccine candidates [31]. As the main immunogenic protein inducing neutralizing antibodies against CSFV, E2 protein is widely used for vaccine production [32]. Secretion of E2 protein is preferred for the large-scale protein production and purification. Studies performed here demonstrated an efficient E2 secretion system in baculovirus relying on the newly identified signal peptide SPZJ. Together with the selected E2 antigen, SPZJ-E2ZJ presents improved immunogenicity and protective immunity against CSFV infection.

Currently E2 subunit vaccines produced in insect cells have not been widely available yet due to the limited yield [33]. Many strategies have been applied to improve E2 expression, including codon optimization of E2 gene in yeast [34] and promoter selection, such as a polyhedrin, p10, and a minimal Drosophila melanogaster Hsp70 promoter to modulate expression [35]. Besides, efforts have been made to signal peptide engineering. Previously, with the deletion of the transmembrane region [36] and the fusion with a certain signal peptide, such as honeybee melittin signal peptide [27] or immunoglobulin kappa (Igκ) signal peptide [37], E2 was adapted to secretion. In this study, compared with a series of truncated signal peptides, results showed that SP23 was important for E2 efficient secretion (Fig. 1b). SPZJ was identified as the most efficient one for E2 secretion in baculovirus system of candidates tested. Besides E2ZJ, SPZJ increase the expression of other E2 types as compared to their native signal peptides (Fig. 2b). E2 yield with SPZJ has been significantly enhanced in comparison to conventional methods. In protein alignment of multiple signal peptides, results indicated that SPZJ shared 86.9% homology with SPC and 95.6% homology with SPHZ (Fig. 2a). 3 amino acids were involved in sequence variations of signal peptides. It was believed that these amino acids be key points in the guidance of protein secretion.

Antigen immunogenicity is mainly determined by protein encoding sequence, which relates to amino acid variation, glycosylation and epitope folding. In IFA, E2ZJ protein induced the higher level of CSFV specific antibodies at the earlier stage than other E2 types tested (Fig. 4a, b), confirming the excellent immunogenicity of E2ZJ. In antigenic regions of E2, E2ZJ of group 2.1 presents several unique mutations which are not found in other strains within the same group. These substitutions in identified or predicted epitopes should be involved in the enhancement in the immunogenicity of E2ZJ against both genotype 1 and 2 CSFV. Besides, proper glycosylation contributes to the improvement in antigen efficacy [38]. E2 glycoprotein contains one putative O-linked glycosylation site (O1) and six N-linked glycosylation sites (N1, N2, N3, N4, N5 and N6) [39]. Among them, O1, N1, N2, N3 and N4 are involved in structural unit assembling, which contributes to the induction of neutralizing antibody [40]. The level of glycosylation of E2ZJ, E2C and E2HZ were detected by glycosylation analyzing algorithm (http://www.cbs.dtu.dk/services/) (data not shown). E2ZJ glycosylation level is between E2C and E2HZ, suggesting the balanced level of glycosylation is favorable for the cross immunogenicity of E2. As all the identified E2 neutralizing epitopes are conserved among variable strains, including E2ZJ [41], the improved immunogenicity of E2ZJ may result from the neighboring variation related to epitope folding for eases in antigen uptake by DCs and B cells. These findings confirmed that with the good immunogenicity, E2ZJ protein serves as a potential candidate vaccine against CSFV infection.

Rational vaccine dosage is critical to determine the vaccine efficacy. Currently, multiple doses are required for most veterinary subunit vaccines and a large amount of antigen in each dose was used to achieve the sufficient efficacy [42]. Due to the incorrect protein folding or modification as the original viral antigens, subunit vaccines produced in prokaryotic systems usually exploit significantly high effective dose. For example, two doses of *E.coli* expressed CAP of 200 μg were required for piglets against PCV2 challenge [43]. 300 μg of E2 from yeast was used to induce neutralizing antibodies after booster immunization against CSFV infection [22]. For other E2 types, booster dose at 40 μg of baculovirus expressed genotype 1.1 and 2.1 E2 was recommended to elicit immune responses against CSFV challenge [18]. However, excessive antigen immunization may cause the tolerance against the target antigen in hosts, leading to the inefficient antibody induction. Besides, the risks of side effects will be much lifted with overdose from other unpredicted ingredients in the vaccine formulation, such as toxin from *E.coli.* Meanwhile, multiple doses at large amounts of antigen will raise the vaccine cost, causing extra economic burden to pig farms. Encouragingly, as shown in the vaccine trials here, a single dose of 5 μg of E2ZJ in piglets is sufficient to elicit complete protection against CSFV lethal challenge. The group of 5 μg-E2ZJ developed sufficient CSFV specific antibodies at 21 dpi and neutralizing antibody at 7 dpi. Previous studies indicated that CSFV specific antibody response from single dose of 32 μg of E2 last for 6 to 13 month after immunization in pigs [21, 44]. In this study, the immunogenic durability of single dose of 5 μg of E2ZJ in pigs was evaluated until 28 dpi, and the protective durability was monitored until 44 dpi (16 dpc, days post challenge). Antibody detection with IDEXX HerdChek® CSFV Kit, together with neutralization test serves as the gold standard for CSFV vaccine efficacy evaluation [18, 22, 45, 46]. In these tests, E2ZJ presents protective immunogenicity, which is comparable to other E2 vaccines. CSFV-1.1 E2, and CSFV-1.1 + 2.1E2 induced specific antibody responses at 28 dpi, with mean antibody blocking rates of 79 and 82%, respectively [18]. 300 μg of yeast expressed E2 induced specific antibody responses at 6 weeks after vaccination with mean antibody blocking rates of 70% and produced complete protection [22]. The antibody blocking rate induced by Marker CSF vaccine rAdV-SFV-E2 based on human adenovirus type 5 was 60% [46]. All these studies reported no detection of viral RNA load in protected pigs after challenge. As the piglets vaccinated with a single dose of 5 μg of E2ZJ developed specific antibody responses at 28 dpi, with mean antibody blocking rates of 79.96% (Fig. 5a) and strong neutralizing immune response, it is believed that CSFV infection was successfully and completely inhibited in E2ZJ pigs, indicating no viral load in protected pigs after challenge. No CSFV related clinical symptoms were observed in any E2ZJ vaccinated piglets upon CSFV challenge and CSFV positive signals were not observed in tissues (Fig. 7b), indicating the sufficient durability of the efficacy of the single dose E2ZJ. Furthermore, neutralization antibodies against both genotype 1 and 2 were detected in all E2ZJ vaccinated animals, confirming the cross protective immunogenicity of E2ZJ. Moreover, for another E2 vaccine candidate (non-ZJE2), which was expressed in HEK293 expression system, one dose of 60 μg failed to induce comparable antibody response as E2ZJ, confirming the advantage of E2ZJ in immunogenicity and vaccine efficacy. Therefore, the single dose of 5 μg of E2ZJ in pigs will be recommended as CSFV vaccine for the complete protection against the disease, which will significantly reduce the cost in vaccination and enhance the vaccine quality.

In summary, the study presented a novel efficient E2 signal peptide for E2 secretion and the lowest effective dose of E2 reported against CSFV lethal challenge in pigs. A single dose of 5 μg of E2ZJ developed a complete protective immune response in pigs and conferred broad protection against the homologous and heterologous CSFV strains. This replies on robust SPZJ-E2ZJ secretion and efficient immunogenicity against CSFV. Hence, serving as a promising vaccine platform, baculovirus expressed SPZJ-E2ZJ is an economical and effective vaccine candidate. It will also be a useful tool for CSF eradication in China, together with other strategies, including differential diagnosis, regional or national-wide eradication campaigns, serological surveillance, and biosecurity procedures.

## Conclusions

The study identified SPZJ (SP23) was characterized to promote strong E2s secretion and significantly improved immunogenicity over conventional counterparts. Besides, a single dose of 5 μg E2ZJ was sufficient to induce protective antibody against CSFV in piglets and conferred broad protection against the homologous and heterologous CSFV strains. The study confirmed that SPZJ-E2ZJ is a cost-effective and efficacious vaccine candidate against CSFV.

## Methods

### Viruses and cells

CSFV strains Shimen (GenBank: FJ598612.1) and HZ08 (GenBank: EF683627) were received from CATG lab, Zhejiang University, China. C-strain was obtained from China Animal Husbandry Industry Co. Ltd.(GenBank: HM175885, Beijing, China). All CSFV strains were propagated in porcine kidney cells (PK-15, ATCC) in Dulbecco’s minimal essential media (DMEM, Hyclone, Thermo Scientific, USA) with 6% fetal bovine serum (FBS, Invitrogen, USA) at 37 °C with 5% CO_2_. High Five and Sf9 insect cells were used to propagate recombinant baculoviruses in SF900 III SFM (Invitrogen, USA) at 27.5 °C.

### Construction of recombinant baculoviruses

The transmembrane domain was deleted from E2. Truncated E2 fragments with a signal peptide (SP) were inserted into the *Eco*RI and *Xho*I sites of the transfer vector pFBD, generating plasmids as pFBD-E2 and pFBD-SP-E2. All plasmids were verified by sequencing analysis. Recombinant baculoviruses Ac-E2 and Ac-SP-E2 were subsequently generated using the Bac-to-Bac system (Invitrogen) according to the manufacturer’s instructions.

### Expression and purification of E2 protein

High Five cells were cultured in 50 ml SF900 III SFM at 27.5 °C with shaking (115 rpm) and then inoculated with recombinant baculoviruses at a multiplicity of infection (MOI) of 1. After 96 h post infection, the culture was centrifuged at 12,000 rpm for 30 min, and supernatant was collected and loaded onto 2 ml Ni-NTA Agarose (Novagen, USA). After washed with 30 ml PBS containing 5 mM imidazole, E2 protein was eluted with 3 ml PBS containing 400 mM imidazole. Purified E2 protein was stored at − 20 °C.

Protein samples were separated on 12% SDS–PAGE gels, transferred to polyvinylidene fluoride (PVDF) membranes (Merck Millipore, USA) and blocked with 5% (w/v) nonfat milk in PBS containing 0.05% Tween (PBST) for 1 h at 37 °C. Membranes were incubated with anti-E2 monoclonal antibody (6D10) (in-house preparation) or GP64 monoclonal antibody (1: 5000 in PBS, Abcam, USA) at 37 °C for 1 h, rinsed with PBST, and incubated with HRP-conjugated goat anti-mouse IgG (1: 5000 in PBS, Sungene, China) at 37 °C for 1 h. After washed with PBST for three times, imunoreactive bands were visualized by Super Signal West Pico/Femto Chemiluminescent Substrate (Thermo Scientific, USA) and images were captured with a Gel 3100 chemiluminescent imaging system (Sage Creation Science).

### Antigen preparation

The concentration of proteins was measured by BCA protein assay kit (Beyotime Biotechnology, China) and the percentage of purified proteins was determined using the layer chromatography scanner (Biotek, USA). The final concentration of target proteins was calculated based on the readings. Antigens were emulsified with ISA-206 adjuvant (Seppic, France) at a ratio of 50:50 (w/w) according to the manufacturer’s instructions.

### Animal immunization and challenge trial

6-week-old female BALB/c mice were purchased from Zhejiang Chinese Medical University Laboratory Animal Research Center (Hangzhou, China). The mice were randomly divided into 4 groups (A, B, C and D, *n* = 5). Groups A to C were respectively immunized twice with a 2-week interval by the subcutaneous injection of 50 μg of E2 (E2ZJ, E2HZ or E2C). Group D was injected with PBS as control. Serum samples were immediately collected on 0, 7, 14, 21 and 28 days post immunization (dpi) for examination of the levels of CSFV-specific antibodies with indirect ELISA and IFA.

Twenty-five piglets were purchased from commercial farm (Hangzhou, China) and were randomly divided into 5 groups (E, F, G, H and L, n = 5) which were free of CSFV before the experiment. Each group was housed individually. Groups E to G were respectively intramuscularly immunized with a single dose of E2ZJ at 5 μg, 15 μg or 30 μg. Group H was intramuscularly immunized with a single dose of 60 μg of Non-ZJ E2. Group L was intramuscularly injected with PBS as control. Serum samples were immediately collected at 0, 7, 21 and 28 days post immunization (dpi) to detect CSFV specific antibodies by commercial ELISA (IDEXX Laboratories, Shiphol-Rijk, The Netherlands) and the neutralizing antibodies against CSFV. At 28 dpi, piglets were intravenously challenged with CSFV Shimen strain (10^5.5^ TCID_50_ in 2 ml PBS), and piglets were checked daily for clinical signs and rectal temperature [47]. All survived animals were euthanized at 16 days post challenge (dpc). Spleens, kidneys, tonsils, lymph nodes and bladders were collected and subjected to pathological examinations [48–50].

### Immunofluorescence assays

PK-15 cells were seeded in 96-well plates 1 day before infection and infected with CSFV strains at MOI of 1. At 48 h post infection, cells were permeabilized with 80% ice-cold acetone in PBS for 30 min at − 20 °C, blocked with 5% (w/v) non-fat milk in PBS for 1 h, and washed once with PBS. Fixed cells were incubated with diluted mouse serum samples (1: 200 in PBS) for 1 h at 37 °C. After washing with PBS for three times, the cells were incubated with FITC-conjugated goat anti-mouse IgG (H + L) (1: 1000 in PBS, Thermo Scientific) as secondary antibody at 37 °C for 1 h. Finally, the labeled cells were treated with the nuclear dye 4′,6′-diamidino-2-phenylindole dihydrochloride (DAPI, 1:2000 dilution in PBS, Beyotime, China). Cells were observed by an inverted fluorescence microscope (Olympus, Corporation, Tokyo, Japan).

### Virus neutralization

Serum samples were twofold serially diluted (starting from 1/4) with DMEM after heat-inactivated for 30 min at 56 °C. Diluted samples were mixed with the equal volume of 100 TCID_50_ of CSFV strain (Shimen or HZ08) and incubated at 37 °C for 1 h. The antibody-virus mixtures were then added to the 96-well plates containing PK-15 cells for 1 h at 37 °C. The highest dilution of serum samples that inhibited virus growth was considered as the neutralization antibody titer and was determined by IFA after incubation in DMEM with 6% FBS at 37 °C for 72 h. IFA was performed as described above.

### Elisa

The presence of CSFV-specific antibodies was determined by either blocking ELISA or indirect ELISA. Blocking ELISA was performed with IDEXX HerdChek® CSFV antibody test kit according to the manufacturer’s instructions. Indirect ELISA was performed according to standard protocols as described previously [51]. Briefly, ELISA plates (Corning, USA) were coated with 100 μl of antigen mixed with E2ZJ, E2HZ, E2Cat a ratio of 1:1:1 (v/v/v) in each well and incubated at 4 °C overnight. The coated plates were then thoroughly washed for three times with PBS containing 0.05% Tween-20 (PBST) and blocked with 5% nonfat milk in PBS for 2 h at 37 °C. Subsequently, Mouse serum samples diluted with PBS containing 5% nonfat milk (1: 200) were added to the plate wells and incubated for 1 h at 37 °C. The plates were washed with PBST for three times and then incubated with 100 μl of HRP-conjugated goat anti-mouse IgG (1: 10000, Sungene, China) diluted with PBS containing 5% nonfat milk for 1 h at 37 °C. After washing with PBST for three times, plates were received 200 μl 3,3′,5,5′ -tetramethylbenzidine (TMB, JingQi Biological, China) at room temperature for 10 min. Finally, 50 μl 2 M H_2_SO_4_ (Sinopharm Cemical Reagent, China) was used to stop the reaction and the absorbance of each well was read at 450 nm using the layer chromatography scanner (Biotek, USA). The mean absorbance value of triplicate wells was used to express CSFV-specific antibodies level.

### Histopathology and immunohistochemistry

Tissue samples (spleen, kidney, submandibular lymphatic nodes and inguinal lymph nodes) were fixed in 4% paraformaldehyde (Sangon, China) at 4 °C for 24 h and embedded in paraffin. Embedded tissues were cut in 5 μm thick sections on a microtome (KuoHai, China). Tissue sections were stained with hematoxylin and eosin (H&E) for pathological evaluation as previously described [46]. Besides, tissues sections were subjected to immunohistochemistry (IHC) using CSFV polyserum (1: 100 in PBS) [52].

### Statistical analysis

Data were expressed as the mean + SD of the three independent experiments. Statistical significance was calculated using a one-way analysis of variance (ANOVA) with multiple comparisons in GraphPad Prism 5 (GraphPad Software, USA). Asterisks *, **, *** or **** in figures indicate statistical significance at the *P* < 0.05, *P* < 0.01, *P* < 0.001 or *P* < 0.0001 level, respectively.

## Data Availability

The datasets used and/or analyzed during the current study are available from the corresponding author on reasonable request. All data generated or analyzed during this study are included in this published article.

## Abbreviations

ATCC: American Type Culture Collection
BCA: Bicinchoninic acid
CSFV: Classical Swine Fever Virus
DAPI: 4′,6′-diamidino-2-phenylindole dihydrochloride
DMEM: Dulbecco’s minimal essential media medium
dpc: Days post challenge
dpi: Days post immunization
ELISA: Enzyme-linked immunosorbent assay
FBS: Fetal bovine serum
FITC: Fluorescein isothiocyanate
H&E: Hematoxylin and eosin
HM: Honeybee melittin
HRP: Horseradish peroxidase
IFA: Indirect immunofluorescence assays
IgG: Immunoglobulin G
IHC: Immunohistochemistry
mAbs: Monoclonal antibodies
MOI: Multiplicity of infection
PBS: Phosphate buffered saline
PBST: PBS containing Tween 20
PK-15: Porcine kidney
PVDF: Polyvinylidene fluoride
SDS-PAGE: Sodium dodecylsulfate-polyacrylamide gel electrophoresis
Sf9: *Spodoptera frugiperda* 9
SP: Signal peptide (SP)
TCID_50_: Tissue culture infective dose
TMB: 3,3′,5,5′ –tetramethylbenzidine
